# Delayed presentation to regular Dutch paediatric care in COVID-19 times: a national survey

**DOI:** 10.1136/bmjpo-2020-000834

**Published:** 2020-10-08

**Authors:** Danielle E M C Jansen, Károly E Illy

**Affiliations:** 1 Department of Health Sciences, University Medical Centre Groningen, Groningen, The Netherlands; 2 Sociology, University of Groningen Faculty of Behavioural and Social Sciences, Groningen, The Netherlands; 3 Ziekenhuis Rivierenland, Tiel, Gelderland, The Netherlands; 4 NVK, Utrecht, The Netherlands

**Keywords:** health services research

## Abstract

We explored the collateral harm in Dutch children and adolescents during the COVID-19 pandemic from experience of paediatricians via an open question distributed via the website of the Dutch Paediatric Society. From the end of March till the first week of July, we received 51 reports of collateral harm involving mostly very young children with mainly acute physical problems but also social problems. In older children, several cases of diabetic ketoacidosis were reported. Our results show that delaying care can lead to seriously ill children, life-threatening situations and that in some cases it can even lead to death. If we want to avoid such a delay at a possible second peak of Corona, general care providers and paediatricians have to join forces and find new ways of working. Systematic data collection of collateral harm in children is needed to be able to intervene adequately.

In their report on the consequences of the corona crisis for regular care, the Dutch Healthcare Authority showed that of all specialisms, paediatrics has experienced the largest decline in the number of urgent referrals in the first weeks of the COVID-19 crisis. In addition, paediatrics was the specialty that showed the least signs of a recovery in referrals (it even fell back slightly) while all other specialties showed a steady recovery from early April onwards.^1^ The decrease in referrals might be explained by a reduced incidence of, for example, traffic and sports injuries as a result of social distancing and school closures. However, the decline might also indicate a delay in care and subsequently in collateral harm. Although some studies report on the consequences of delayed presentations to emergency departments, insight in the nature and severity of delayed presentation to regular paediatric care is missing.^2 3^ Our objective was to explore the collateral harm in Dutch children and adolescents during the COVID-19 pandemic from the experience of paediatricians.

Via the website of the Dutch Paediatric Society, we requested all 1400 paediatricians affiliated with the professional association (93% of all Dutch paediatricians) to report on collateral harm in children and adolescents, from 2 weeks since the initiation of the Dutch ‘intelligent lockdown’,^4^ a lighter version of a full lockdown^5^ (end of March) to the first week of July. The question was: ‘We ask you to report if, in your opinion, a child was presented too late to acute, regular or chronic care due to parental or healthcare provider concerns about corona, and which resulted in unnecessary harm’.

The results of this inventory showed 51 reports (from 38 respondents divided over 31 hospitals) of collateral harm since the end of March. Although the majority of reports of harm were received in the first 4 weeks of the intelligent lockdown (n=27), there were still 24 reports in the months after, up to and including the second week of July. The reports came from all over the Netherlands, but most reports were received from the west and south-west of the Netherlands, the regions where the corona crisis was more severe and, in all likelihood, experienced the highest pressure on healthcare.

Most reports (54%) of collateral harm involved young children: neonates, infants and children aged 1–4 years (table 1). The symptoms with which the children were presented—too late—to the paediatrician varied widely, including mainly acute physical problems but also social problems. The delay in neonatal care was mainly related to hyperbilirubinaemia and weight-related problems (low birth weight and severe weight loss). Spread across multiple age groups, several children presented with diabetic ketoacidosis (table 2).

**Table 1:** Background characteristics of cases (n=51)

Background characteristics	
Gender	Male: 9 Female: 11 Unknown: 29
Age of reported cases	Neonate (under 28 days of age): 9 (18%) Infant (under 1 year of age): 10 (20%) Aged 1–4 years: 8 (16%) Aged 4–12 years: 5 (10%) Aged 12–16 years: 6 (12%) Unknown/not reported: 13 (26%)
Province of report (location of province and no/% of registered patients with COVID-19 on 24 March 2020)*	South Holland (West Netherlands; 758 (13.6%)): 10 North Holland (West Netherlands; 740 (13.3%)): 9 North Brabant (South Netherlands; 1739 (31.3%)): 9 Gelderland (East Netherlands; 593 (10.7%)): 6 Utrecht (West Netherlands; 478 (8.6%)): 4 Limburg (South Netherlands; 690 (12.4%)): 3 Drenthe (North Netherlands; 55 (1%)): 2 Friesland (North Netherlands; 42 (0.8%)): 2 Zeeland (West Netherlands; 63 (1.1%)): 2 Groningen (North Netherlands; 71 (1.3%)): 1 Overijssel (East Netherlands; 257 (4.4%)): 1 Flevoland (East Netherlands; 74 (1.3%)): 0

*https://www.rivm.nl/sites/default/files/2020-03/Epidemiologische_situatie_COVID-19_24_maart_2020.pdf.

**Table 2:** Reports of collateral harm

Paediatric subspecialty ↓	Collateral harm →
Cardiology	Broadened mediastinum with a vena cava superior syndrome, due to a lymphoma Complex cor vitium Critical pulmonary stenosis and right ventricular hypertrophy with poor right ventricle dysfunction Congenital cyanotic heart disease
Child abuse paediatrics	Died due to serious abuse Impending out of home placement Oppression of the brains due to subdural hematoma
Ear, nose and throat	Extensive soft-tissue swelling in the mouth due to abscess
Endocrinology	Diabetes mellitus de novo (1×) Diabetes mellitus de novo with severe diabetic ketoacidosis 3× Diabetic ketoacidosis (5×)
Gastroenterology and nutrition	Abscess in the abdomen after appendicitis (3×) Developmental delay due to carnitine deficiency Low birth weight (2×) Oral aversion Severe weight loss Severe dehydration with hypochloraemic alkalosis, hypokalemia and hyponatremia Vitamin B_12_ and folic acid deficiency
Genetics and metabolic diseases	Long-term breathing stop and diarrhoea in child with Cockayne syndrome
Haematology	Anaemia with signs of impending circulatory insufficiency Hyperbilirubinaemia (2×)
Infectious diseases	A-typical COVID-19 symptoms Impetigo bullosa and suspected Staphylococcal Scalded Skin Syndrome (SSSS) Infected, necrotic varicella lesions Kawasaki-like symptoms Mastoiditis Sepsis Shock due to group B streptococcal septicaemia
Neurology	Severe neurological complication after manual therapy
Oncology	Leukaemia Persistent fever and suspicion of lymphoma Possible benign tumour from tonsil/uvula
Pulmonology	Asphyxia Asthma Respiratory failure with respiratory infection Subglottic stenosis, due to haemangioma
Psychiatry	Anorexia Severe weight loss, a. mesenteric superior syndrome, acute renal failure, ulcers, leucopenia with fever, traumatic injury after attempted suicide
Other	Unexplained clinical deterioration Insufficient follow-up ex-premature

Although the results of this exploration among paediatricians shows an alarming situation, this is probably only the tip of the iceberg since it is an exploration in which the data were not collected systematically. Our results show that delaying care can lead to seriously ill children, life-threatening situations and that in some cases it can even lead to death. If we want to avoid such a delay in providing the right care at the right place by the right person, at a possible second peak of Corona, action must be taken in which general care providers and paediatricians have to join forces, in particular regarding triage. We need to find new ways of working for unusual times like this so that the delay in care is avoided at all times. Finally, there should be systematic data collection of collateral harm in children; this is the only way to clarify its causes so that targeted interventions can be made.

## Supplementary Material

Author's manuscript
