# Supplementary material for: Delayed presentation to regular Dutch paediatric care in COVID-19 times: a national survey
Source: BMJ Paediatr Open. 2020 Oct 8;4(1):e000834. doi: 10.1136/bmjpo-2020-000834 (PMC7545654; doi:10.1136/bmjpo-2020-000834)
Supplement: Author's manuscript [file bmjpo-2020-000834.draft_revisions.pdf]

BMJ Paediatrics Open

BMJ Paediatrics Open is committed to open peer review. As part of this commitment we make the peer review history of every article we publish publicly available.

When an article is published we post the peer reviewers' comments and the authors' responses online. We also post the versions of the paper that were used during peer review. These are the versions that the peer review comments apply to.

The versions of the paper that follow are the versions that were submitted during the peer review process. They are not the versions of record or the final published versions. They should not be cited or distributed as the published version of this manuscript.

BMJ Paediatrics Open is an open access journal and the full, final, typeset and author-corrected version of record of the manuscript is available on our site with no access controls, subscription charges or pay-per-view fees (http://bmjpaedsopen.bmj.com).

If you have any questions on BMJ Paediatrics Open's open peer review process please email info.bmjpo@bmj.com

BMJ Paediatrics Open

Delayed presentation to regular Dutch paediatric care in COVID-19 times: a national survey.

Journal:	*BMJ Paediatrics Open*
Manuscript ID	bmjpo-2020-000834
Article Type:	Original research letter
Date Submitted by the Author:	08-Aug-2020

Complete List of Authors:	Jansen, Danielle; University Medical Centre Groningen, Department of Health Sciences; University of Groningen Faculty of Behavioural and Social Sciences, Sociology<br>Illy, Károly; Ziekenhuis Rivierenland; NVK

Keywords:	Health services research

SCHOLARONE™
Manuscripts

I, the Submitting Author has the right to grant and does grant on behalf of all authors of the Work (as defined in the below author licence), an exclusive licence and/or a non-exclusive licence for contributions from authors who are: i) UK Crown employees; ii) where BMJ has agreed a CC-BY licence shall apply, and/or iii) in accordance with the terms applicable for US Federal Government officers or employees acting as part of their official duties; on a worldwide, perpetual, irrevocable, royalty-free basis to BMJ Publishing Group Ltd ("BMJ") its licensees and where the relevant Journal is co-owned by BMJ to the co-owners of the Journal, to publish the Work in this journal and any other BMJ products and to exploit all rights, as set out in our licence.

The Submitting Author accepts and understands that any supply made under these terms is made by BMJ to the Submitting Author unless you are acting as an employee on behalf of your employer or a postgraduate student of an affiliated institution which is paying any applicable article publishing charge ("APC") for Open Access articles. Where the Submitting Author wishes to make the Work available on an Open Access basis (and intends to pay the relevant APC), the terms of reuse of such Open Access shall be governed by a Creative Commons licence – details of these licences and which Creative Commons licence will apply to this Work are set out in our licence referred to above.

Other than as permitted in any relevant BMJ Author's Self Archiving Policies, I confirm this Work has not been accepted for publication elsewhere, is not being considered for publication elsewhere and does not duplicate material already published. I confirm all authors consent to publication of this Work and authorise the granting of this licence.

Confidential: For Review Only

Delayed presentation to regular Dutch paediatric care in COVID-19 times: a national survey.

Jansen DEMC[1,2] & Illy KE[3,4]

[1] Department of Health Sciences, University Medical Center Groningen, University of Groningen, Groningen, the Netherlands.
[2] Department of Sociology and Interuniversity Center for Social Science Theory and Methodology (ICS), University of Groningen, Groningen, the Netherlands.
[3] Hospital Rivierenland, Tiel, the Netherlands.
[4] Dutch Paediatric Society, Utrecht, the Netherlands.

Corresponding author:
DEMC Jansen, PhD
University Medical Center Groningen, University of Groningen, Department of Health Sciences
Antonius Deusinglaan 1, FA10, Room 4.06
9713 AV Groningen
Telephone: +3150 3616675 36
The Netherlands
Email: d.e.m.c.jansen@umcg.nl

Confidential: For Review Only

Abstract

We explored the collateral damage in Dutch children and adolescents during the COVID-19 pandemic from experience of paediatricians via an open question distributed via the website of the Dutch Paediatric Society. From the end of March till the first week of July we received 49 reports of collateral damage involving mostly very young children with mainly acute physical problems but also social problems. In older children several cases of diabetic ketoacidosis were reported. Our results show that delaying care can lead to seriously ill children, life-threatening situations and that in some cases it can even lead to death. If we want to avoid such a delay at a possible second peak of Corona, general care providers and paediatricians have to join forces and find new ways of working. Systematic data collection of collateral damage in children is needed to be able to intervene adequately.

In their report on the consequences of the corona crisis for regular care, the Dutch Healthcare Authority showed that of all specialisms, paediatrics has experienced the largest decline in the number of urgent referrals in the first weeks of the COVID-19 crisis. In addition, paediatrics was the specialism that showed the least signs of a recovery in referrals (it even fell back slightly) while all other specialisms showed a steady recovery from early April onwards.[1] This decline in referrals might indicate a delay in care and subsequently in collateral damage. Although some studies report on the consequences of delayed presentations to hospital pediatric emergency departments, insight in the nature and severity of delayed presentation to regular pediatric care is missing.[2,3] Our objective was to explore the collateral damage in Dutch children and adolescents during the COVID-19 pandemic from the experience of paediatricians.

Via the website of the Dutch Paediatric Society, we requested all 1400 paediatricians affiliated with the professional association (93% of all Dutch paediatricians) to report on collateral damage in children and adolescents, from two weeks since the initiation of the Dutch “intelligent lockdown”, a lighter version of a full lock-down (end of March) to the first week of July. The question was: “We ask you to report if, in your opinion, a child was presented too late to acute, regular or chronic care due to parental or health care provider concerns about corona, and which resulted in unnecessary damage.”

The results of this inventory showed a very worrying picture with 51 reports of collateral damage since the end of March. Although the majority of reports of damage were received in the first weeks of the intelligent lockdown (n=27), there were still 24 reports in the months after, up to and including the second week of July. The reports came from all over the Netherlands, but most reports were received from the west and south-west of the Netherlands, the regions where the corona crisis was more severe and, in all likelihood, experienced the highest pressure on healthcare.

Most reports (54%) of collateral damage involved young children: neonates, infants and children aged 1 to 4 years (table 1). The symptoms with which the children were presented - too late - to the paediatrician varied widely, including mainly acute physical problems but also social problems. The delay in neonatal care was mainly related to hyperbilirubinemia and weight-related problems (low birth weight and severe weight loss). Spread across multiple age groups, several children presented with diabetic ketoacidosis.

Confidential: For Review Only

Although the results of this exploration among paediatricians shows an alarming situation, this is probably only the tip of the iceberg since it is an exploration in which the data were not collected systematically. However, our results show that delaying care can lead to seriously ill children, life-threatening situations and that in some cases it can even lead to death. If we want to avoid such a delay in providing the right care at the right place by the right person, at a possible second peak of Corona, action must be taken in which general care providers and paediatricians have to join forces, in particular regarding triage. We need to find new ways of working for unusual times like this so that the delay in care is avoided at all times. Finally, there should be systematic data collection of collateral damage in children; this is the only way to clarify its causes so that targeted interventions can be made.

Competing interests: None declared.

Patient and public involvement: Patients and/or the public were not involved in the design, or conduct, or reporting, or dissemination plans of this research.

Patient consent for publication: Not required.

1 Dutch Healthcare Authority. Analysis of the consequences of the corona crisis for regular care. 2020. https://puc.overheid.nl/nza/doc/PUC_307166_22/1/ (accessed July 13, 2020).
2 Ciacchini B, Tonioli F, Marciano C, Faticato MG, Borali E, Pini Prato A and E Felici. Reluctance to seek pediatric care during the COVID-19 pandemic and the risks of delayed diagnosis. *Italian Journal of Pediatrics* (2020) 46:87.
3 Roland D, Harwood R, Bishop N, Hargreaves D, Patel S and I Sinha. Children's emergency presentations during the COVID-19 pandemic. *The Lancet Child & Adolescent Health* (2020) 4: 8.

Confidential: For Review Only

Table 1: Background characteristics of cases (n=51)

**Background characteristics**	
Gender	Male: 9<br>Female: 11<br>Unknown: 29
Age of reported cases	-9 months: 1 (2%)<br>Neonate (under 28 days of age): 8 (16%)<br>Infant (under one year of age): 10 (20%)<br>Aged 1 to 4 years: 8 (16%)<br>Aged 4 to 12 years: 5 (10%)<br>Aged 12 to 16 years: 6 (12%)<br>Unknown/not reported: 13 (26%)
Province of report	South Holland: 10<br>North Holland: 9<br>Brabant: 9<br>Gelderland: 6<br>Utrecht: 4<br>Limburg: 3<br>Drenthe: 2<br>Friesland: 2<br>Zeeland: 2<br>Groningen: 1<br>Overijssel: 1

Confidential: For Review Only

Table 2: reports of collateral damage.

Paediatric subspecialty ↓	Collateral damage →
Cardiology	- Broadened mediastinum with a vena cava superior syndrome, due to a lymphoma<br>- Complex cor vitium<br>- Critical pulmonary stenosis and right ventricular hypertrophy with poor right ventricle dysfunction<br>- Congenital cyanotic heart disease
Child Abuse Paediatrics	- Died due to serious abuse<br>- Impending out of home placement<br>- Oppression of the brains due to subdural hematoma
Ear, Nose and Throat	- Extensive soft tissue swelling in the mouth due to abscess
Endocrinology	- Diabetes mellitus de novo (1x)<br>- Diabetes mellitus de novo with severe diabetic ketoacidosis 3x<br>- Diabetic ketoacidosis (5x)
Gastroenterology and nutrition	- Abscess in the abdomen after appendicitis (3x)<br>- Developmental delay due to carnitine deficiency<br>- Low birth weight (2x)<br>- Oral aversion<br>- Severe weight loss<br>- Severe dehydration with hypochloremic alkalosis, hypokalemia and hyponatremia<br>- Vitamin B12 and folic acid deficiency
Genetics and Metabolic Diseases	- Long-term breathing stop and diarrhea in child with Cockayne syndrome
Haematology	- Anaemia with signs of impending circulatory insufficiency<br>- Hyperbilirubinemia (2x)
Infectious diseases	- A-typical COVID-19 symptoms<br>- Impetigo bullosa and suspected Staphylococcal Scalded Skin Syndrome (SSSS)<br>- Infected, necrotic varicella lesions<br>- Kawasaki-like symptoms<br>- Mastoiditis<br>- Sepsis<br>- Shock due to group B streptococcal septicaemia
Neurology	- Severe neurological complication after manual therapy
Oncology	- Leukaemia<br>- Persistent fever and suspicion of lymphoma<br>- Possible benign tumour from tonsil/uvula
Pulmonology	- Asphyxia<br>- Asthma<br>- Respiratory failure with respiratory infection<br>- Subglottic stenosis, due to haemangioma

Confidential: For Review Only

Paediatric subspecialty ↓	Collateral damage →
Psychiatry	- Anorexia<br>- Severe weight loss, a. mesenteric superior syndrome, acute renal failure, ulcers, leukopenia with fever, traumatic injury after attempted suicide
Other	- Unexplained clinical deterioration<br>- Insufficient follow-up ex-premature

Confidential: For Review Only

BMJ Paediatrics Open

Delayed presentation to regular Dutch paediatric care in COVID-19 times: a national survey.

Journal:	*BMJ Paediatrics Open*
Manuscript ID	bmjpo-2020-000834.R1
Article Type:	Original research letter
Date Submitted by the Author:	17-Sep-2020
Complete List of Authors:	Jansen, Danielle; University Medical Centre Groningen, Department of Health Sciences; University of Groningen Faculty of Behavioural and Social Sciences, Sociology<br>Illy, Károly; Ziekenhuis Rivierenland; NVK
Keywords:	Health services research

SCHOLARONE™
Manuscripts

I, the Submitting Author has the right to grant and does grant on behalf of all authors of the Work (as defined in the below author licence), an exclusive licence and/or a non-exclusive licence for contributions from authors who are: i) UK Crown employees; ii) where BMJ has agreed a CC-BY licence shall apply, and/or iii) in accordance with the terms applicable for US Federal Government officers or employees acting as part of their official duties; on a worldwide, perpetual, irrevocable, royalty-free basis to BMJ Publishing Group Ltd ("BMJ") its licensees and where the relevant Journal is co-owned by BMJ to the co-owners of the Journal, to publish the Work in this journal and any other BMJ products and to exploit all rights, as set out in our licence.

The Submitting Author accepts and understands that any supply made under these terms is made by BMJ to the Submitting Author unless you are acting as an employee on behalf of your employer or a postgraduate student of an affiliated institution which is paying any applicable article publishing charge ("APC") for Open Access articles. Where the Submitting Author wishes to make the Work available on an Open Access basis (and intends to pay the relevant APC), the terms of reuse of such Open Access shall be governed by a Creative Commons licence – details of these licences and which Creative Commons licence will apply to this Work are set out in our licence referred to above.

Other than as permitted in any relevant BMJ Author's Self Archiving Policies, I confirm this Work has not been accepted for publication elsewhere, is not being considered for publication elsewhere and does not duplicate material already published. I confirm all authors consent to publication of this Work and authorise the granting of this licence.

Confidential: For Review Only

Delayed presentation to regular Dutch paediatric care in COVID-19 times: a national survey.

Jansen DEMC[1,2] & Illy KE[3,4]

[1] Department of Health Sciences, University Medical Center Groningen, University of Groningen, Groningen, the Netherlands.
[2] Department of Sociology and Interuniversity Center for Social Science Theory and Methodology (ICS), University of Groningen, Groningen, the Netherlands.
[3] Hospital Rivierenland, Tiel, the Netherlands.
[4] Dutch Paediatric Society, Utrecht, the Netherlands.

Word count: 600

Corresponding author:
DEMC Jansen, PhD
University Medical Center Groningen, University of Groningen, Department of Health Sciences
Antonius Deusinglaan 1, FA10, Room 4.06
9713 AV Groningen
Telephone: +3150 3616675 36
The Netherlands
Email: d.e.m.c.jansen@umcg.nl

Confidential: For Review Only

Abstract
We explored the collateral harm in Dutch children and adolescents during the COVID-19 pandemic from experience of paediatricians via an open question distributed via the website of the Dutch Paediatric Society. From the end of March till the first week of July we received 49 reports of collateral harm involving mostly very young children with mainly acute physical problems but also social problems. In older children several cases of diabetic ketoacidosis were reported. Our results show that delaying care can lead to seriously ill children, life-threatening situations and that in some cases it can even lead to death. If we want to avoid such a delay at a possible second peak of Corona, general care providers and paediatricians have to join forces and find new ways of working. Systematic data collection of collateral harm in children is needed to be able to intervene adequately.

In their report on the consequences of the corona crisis for regular care, the Dutch Healthcare Authority showed that of all specialisms, paediatrics has experienced the largest decline in the number of urgent referrals in the first weeks of the COVID-19 crisis. In addition, paediatrics was the specialty that showed the least signs of a recovery in referrals (it even fell back slightly) while all other specialties showed a steady recovery from early April onwards.[1] The decrease in referrals might be explained by a reduced incidence of e.g. traffic and sports injuries as a result of social distancing and school closures. However, the decline might also indicate a delay in care and subsequently in collateral harm. Although some studies report on the consequences of delayed presentations to emergency departments, insight in the nature and severity of delayed presentation to regular pediatric care is missing.[2,3] Our objective was to explore the collateral harm in Dutch children and adolescents during the COVID-19 pandemic from the experience of paediatricians.

Via the website of the Dutch Paediatric Society, we requested all 1400 paediatricians affiliated with the professional association (93% of all Dutch paediatricians) to report on collateral harm in children and adolescents, from two weeks since the initiation of the Dutch "intelligent lockdown"[4], a lighter version of a full lock-down[5] (end of March) to the first week of July. The question was: "We ask you to report if, in your opinion, a child was presented too late to acute, regular or chronic care due to parental or health care provider concerns about corona, and which resulted in unnecessary harm."

The results of this inventory showed 51 reports (from 38 respondents divided over 31 hospitals) of collateral harm since the end of March. Although the majority of reports of harm were received in the first four weeks of the intelligent lockdown (n=27), there were still 24 reports in the months after, up to and including the second week of July. The reports came from all over the Netherlands, but most reports were received from the west and south-west of the Netherlands, the regions where the corona crisis was more severe and, in all likelihood, experienced the highest pressure on healthcare.

Most reports (54%) of collateral harm involved young children: neonates, infants and children aged 1 to 4 years (table 1). The symptoms with which the children were presented - too late - to the paediatrician varied widely, including mainly acute physical problems but also social problems. The delay in neonatal care was mainly related to hyperbilirubinemia and weight-related problems (low birth weight and severe weight loss). Spread across multiple age groups, several children presented with diabetic ketoacidosis.

Although the results of this exploration among paediatricians shows an alarming situation, this is probably only the tip of the iceberg since it is an exploration in which the data were not collected systematically. Our results show that delaying care can lead to seriously ill children, life-threatening situations and that in some cases it can even lead to death. If we want to avoid such a delay in providing the right care at the right place by the right person, at a possible second peak of Corona, action must be taken in which general care providers and paediatricians have to join forces, in particular regarding triage. We need to find new ways of working for unusual times like this so that the delay in care is avoided at all times. Finally, there should be systematic data collection of collateral harm in children; this is the only way to clarify its causes so that targeted interventions can be made.

Contributors: DJ drafted the initial manuscript. KI provided significant contributions to the data collection that led to this manuscript. DJ and KI provided significant contributions to the data analysis and presentation used in this manuscript. KI critically reviewed and revised the manuscript.

Funding: This research received no specific grant from any funding agency in the public, commercial or not-for-profit sectors.

Competing interests: None declared.

Patient and public involvement: Patients and/or the public were not involved in the design, or conduct, or reporting, or dissemination plans of this research.

Patient consent for publication: Not required.

1 Dutch Healthcare Authority. Analysis of the consequences of the corona crisis for regular care. 2020. https://puc.overheid.nl/nza/doc/PUC_307166_22/1/ (accessed July 13, 2020).
2 Ciacchini B, Tonioli F, Marciano C, Faticato MG, Borali E, Pini Prato A and E Felici. Reluctance to seek pediatric care during the COVID-19 pandemic and the risks of delayed diagnosis. *Italian Journal of Pediatrics* (2020) 46:87.
3 Roland D, Harwood R, Bishop N, Hargreaves D, Patel S and I Sinha. Children's emergency presentations during the COVID-19 pandemic. *The Lancet Child & Adolescent Health* (2020) 4: 8.
4 https://en.wikipedia.org/wiki/COVID-19_pandemic_in_the_Netherlands
5 De Haas M, Faber R, Hamersma M, How COVID-19 and the Dutch 'intelligent lockdown' change activities, work and travel behaviour: Evidence from longitudinal data in the Netherlands, *Transportation Research Interdisciplinary Perspectives*, 6, 2020, https://doi.org/10.1016/j.trip.2020.100150.

Confidential: For Review Only

Table 1: Background characteristics of cases (n=51)

**Background characteristics**	
Gender	Male: 9<br>Female: 11<br>Unknown: 29
Age of reported cases	Neonate (under 28 days of age): 9 (18%)<br>Infant (under one year of age): 10 (20%)<br>Aged 1 to 4 years: 8 (16%)<br>Aged 4 to 12 years: 5 (10%)<br>Aged 12 to 16 years: 6 (12%)<br>Unknown/not reported: 13 (26%)
Province of report (location of province and no/% of registered COVID-19 patients on 24 March 2020)[1]	South Holland (West Netherlands; 758 (13.6%)): 10<br>North Holland (West Netherlands; 740 (13.3%)): 9<br>North Brabant (South Netherlands; 1739 (31.3%)): 9<br>Gelderland (East Netherlands; 593 (10.7%)): 6<br>Utrecht (West Netherlands; 478 (8.6%)): 4<br>Limburg (South Netherlands; 690 (12.4%)): 3<br>Drenthe (North Netherlands; 55 (1%)): 2<br>Friesland (North Netherlands; 42 (0.8%)): 2<br>Zeeland (West Netherlands; 63 (1.1%)): 2<br>Groningen (North Netherlands; 71 (1.3%)): 1<br>Overijssel (East Netherlands; 257 (4.4%)): 1<br>Flevoland (East Netherlands; 74 (1.3%)): 0

[1] https://www.rivm.nl/sites/default/files/2020-03/Epidemiologische%20situatie%20COVID-19%2024%20maart%202020.pdf

Confidential: For Review Only

Table 2: reports of collateral harm.

Paediatric subspecialty ↓	Collateral harm →
Cardiology	- Broadened mediastinum with a vena cava superior syndrome, due to a lymphoma<br>- Complex cor vitium<br>- Critical pulmonary stenosis and right ventricular hypertrophy with poor right ventricle dysfunction<br>- Congenital cyanotic heart disease
Child Abuse Paediatrics	- Died due to serious abuse<br>- Impending out of home placement<br>- Oppression of the brains due to subdural hematoma
Ear, Nose and Throat	- Extensive soft tissue swelling in the mouth due to abscess
Endocrinology	- Diabetes mellitus de novo (1x)<br>- Diabetes mellitus de novo with severe diabetic ketoacidosis 3x<br>- Diabetic ketoacidosis (5x)
Gastroenterology and nutrition	- Abscess in the abdomen after appendicitis (3x)<br>- Developmental delay due to carnitine deficiency<br>- Low birth weight (2x)<br>- Oral aversion<br>- Severe weight loss<br>- Severe dehydration with hypochloremic alkalosis, hypokalemia and hyponatremia<br>- Vitamin B12 and folic acid deficiency
Genetics and Metabolic Diseases	- Long-term breathing stop and diarrhea in child with Cockayne syndrome
Haematology	- Anaemia with signs of impending circulatory insufficiency<br>- Hyperbilirubinemia (2x)
Infectious diseases	- A-typical COVID-19 symptoms<br>- Impetigo bullosa and suspected Staphylococcal Scalded Skin Syndrome (SSSS)<br>- Infected, necrotic varicella lesions<br>- Kawasaki-like symptoms<br>- Mastoiditis<br>- Sepsis<br>- Shock due to group B streptococcal septicaemia
Neurology	- Severe neurological complication after manual therapy
Oncology	- Leukaemia<br>- Persistent fever and suspicion of lymphoma<br>- Possible benign tumour from tonsil/uvula
Pulmonology	- Asphyxia

Confidential: For Review Only

Paediatric subspecialty ↓	Collateral harm →
	- Asthma<br>- Respiratory failure with respiratory infection<br>- Subglottic stenosis, due to haemangioma
Psychiatry	- Anorexia<br>- Severe weight loss, a. mesenteric superior syndrome, acute renal failure, ulcers, leukopenia with fever, traumatic injury after attempted suicide
Other	- Unexplained clinical deterioration<br>- Insufficient follow-up ex-premature

Confidential: For Review Only
